# Dot Display Affects Approximate Number System Acuity and Relationships with Mathematical Achievement and Inhibitory Control

**DOI:** 10.1371/journal.pone.0155543

**Published:** 2016-05-19

**Authors:** Jade Eloise Norris, Julie Castronovo

**Affiliations:** 1 Department of Psychology, Swansea University, Singleton Park, Swansea, SA2 8PP, Wales; 2 Department of Psychology, University of Hull, Cottingham Road, Hull, HU6 7RX, England; University of Leuven, BELGIUM

## Abstract

Much research has investigated the relationship between the Approximate Number System (ANS) and mathematical achievement, with continued debate surrounding the existence of such a link. The use of different stimulus displays may account for discrepancies in the findings. Indeed, closer scrutiny of the literature suggests that studies supporting a link between ANS acuity and mathematical achievement in adults have mostly measured the ANS using spatially intermixed displays (e.g. of blue and yellow dots), whereas those failing to replicate a link have primarily used spatially separated dot displays. The current study directly compared ANS acuity when using intermixed or separate dots, investigating how such methodological variation mediated the relationship between ANS acuity and mathematical achievement. ANS acuity was poorer and less reliable when measured with intermixed displays, with performance during both conditions related to inhibitory control. Crucially, mathematical achievement was significantly related to ANS accuracy difference (accuracy on congruent trials minus accuracy on incongruent trials) when measured with intermixed displays, but not with separate displays. The findings indicate that methodological variation affects ANS acuity outcomes, as well as the apparent relationship between the ANS and mathematical achievement. Moreover, the current study highlights the problem of low reliabilities of ANS measures. Further research is required to construct ANS measures with improved reliability, and to understand which processes may be responsible for the increased likelihood of finding a correlation between the ANS and mathematical achievement when using intermixed displays.

## Introduction

The ‘number sense’, also known as the Approximate Number System [1], is a foundational ability used to process non-symbolic numerical magnitudes (e.g. sets of dots) without counting. This innate system, originating from primitive, evolutionary functions is observed in infants, children, adults, and even some animals [2–4]. The number sense obeys Weber’s law, as indicated by size effects (slower and less accurate processing of numerosity as size increases) and ratio effects (faster and more accurate discrimination between numerosities as their ratio increases [4,5]). ANS acuity is commonly measured with the Weber fraction [1], a value between 0 and 1 [2] which indicates the amount of noise present in an individual’s numerosity representations, with a higher *w* representing a noisier, less accurate representation [1]. ANS acuity is also frequently measured by accuracy and reaction times [6], ratio effects [7], point of subjective equality [8], or a mix of these measures [1,9,10]. Each measure has been implicitly assumed to index the ANS [11]. However, when directly comparing the reliability of each measure, Inglis and Gilmore [11] found accuracy to be a superior index of ANS acuity over *w*, with numerical ratio effects (NREs) cited as the least reliable measure.

As well as measures indexing ANS acuity, experimental methods used to test the ANS have varied widely [12]. Comparison tasks are most frequently used to measure ANS acuity, briefly displaying two sets of non-symbolic stimuli (e.g. dots) on a computer screen, with participants deciding approximately which set is more numerous. Alternatives, such as change detection paradigms, have been used to accommodate participant age (e.g. infants [13]). Studies using these varying experimental designs are then directly compared in the literature to discuss the development of the ANS from early childhood to adulthood. Such task variation may therefore present a problem when assessing the lifespan development of the number sense, as it is unclear whether changes reflect ANS acuity development or in fact reveal methodological differences [7,10,14,15]. This suggestion is supported by research concluding that *w*s calculated for performance on same/different, habituation, and comparison tasks are poorly correlated, with superior accuracy during comparison tasks for both children and adults [14,16]. It is clear that comparison tasks cannot practicably be used to measure ANS acuity in infants. However, for the majority of ANS acuity research beyond infant populations, inconsistencies in task type [17], stimulus display, number of trials [18], set size [19], perceptual variable control, acuity measures (RTs, accuracy, NRE and *w*), and display times may lead to misrepresentation of ANS acuity [20] and its links with other abilities, including mathematical achievement [11,15,21].

In testing the relationship between mathematical achievement and the ANS in adults, the majority of studies finding a significant correlation have used spatially intermixed dot displays in non-symbolic comparison tasks ([22–24], but see [25]) whereas those finding no correlation have most often used spatially separated [26–30] or sequentially presented displays [9]. Therefore, stimulus presentation differences may drive the presence or otherwise of a correlation between ANS acuity and mathematical achievement in adults [11,15]. Considering that recent research has begun to uncover evidence that training the ANS may improve mathematical achievement [31], that dyscalculia is indicated by ANS deficits [32], and most notably that ANS acuity predicts mathematical ability across the lifespan [24], examination of the impact of methodological variation on ANS acuity is important in reaching reliable conclusions about the ANS and its relationship with mathematical achievement.

An important factor currently under considerable debate is the contribution of inhibitory control to numerosity judgements during an ANS task. Dot size and surface area controls have been included to ensure that participants make numerosity judgements, rather than judgements based on other perceptual variables. Methods used to control the influence of perceptual variables during numerosity comparison tasks have varied [19,33], with most using congruent trials (dot area and numerosity correlate, so that the more numerous set has a larger cumulative area and as a consequence a larger average dot size) and incongruent trials (cumulative area is matched between sets, resulting in a larger dot size on average in the least numerous set). Such designs are suggested to present a Stroop-like effect [10,26,34], as during ‘incongruent’ trials the irrelevant and incongruent dimension (dot size) must be inhibited to judge the relevant dimension (numerosity). Further, some authors have concluded that ANS tasks may predominantly measure inhibitory abilities, and that correlations between mathematical achievement and the ANS may primarily reflect a relationship between inhibition and mathematical achievement rather than a correlation between ANS acuity and mathematical achievement as such [6,19,26]. The effect of dot size and cumulative area controls will therefore be further investigated in the current study using congruent and incongruent trials.

The current study aims to investigate the effect of separate or intermixed dot displays on ANS acuity using the ‘Panamath’ software (www.panamath.org/researchers: [1]). A previous study has attempted to address this methodological issue [15]. Price et al. (2012) used a within-subjects design to investigate the impact of using spatially intermixed, separated, or sequential dot displays on ANS acuity, finding similar performance across conditions. However, stimuli were visible for 750ms which may have encouraged counting, potentially facilitating performance in the more difficult conditions, therefore disguising any ANS acuity differences between display formats. Further, the authors used *w* and NREs as dependent variables for ANS acuity, with the latter suggested as an unreliable index of the ANS [11]. Finally, although a within-subjects design allowed for direct comparison between participants’ performances on each condition, that participants were exposed to hundreds of numerosity comparison trials may have enhanced their familiarity with the task, reducing differences between performances on each condition. The current study will therefore use a between-subjects design to examine ANS acuity during congruent and incongruent trials (*w*, accuracy, and RTs) derived from intermixed or separated displays. The link between ANS acuity as measured by different dot displays and mathematical achievement will also be investigated. Further, inhibition will be measured to examine the relationship between inhibitory control and ANS task performance using a colour Stroop [35] and a number Stroop task [36]. It is predicted that intermixed displays will result in poorer ANS acuity (higher *w*s, lower accuracy, and slower RTs) due to the extra step required in resolving the overlapping dot arrays [10] prior to processing numerosity. Further, it is predicted that a relationship between mathematical achievement and ANS acuity will be predominantly evident in the intermixed condition, reflecting the pattern of results from the current literature.

## Method

### Participants

Sixty-four participants aged 18–25 (24 males, M_age_ = 19.8, SD = 1.68) were recruited through the Department of Psychology at the University of Hull. Participants received course credit for participation. The study gained ethical approval from the University Of Hull Department Of Psychology Ethics Committee. All participants were fully informed of the study aims and provided written, informed consent. Participants were screened at recruitment for a history of psychiatric disorder, depression, and abnormal vision.

### Measures

#### Spelling

Part 2 of the Wide Range Achievement Test 4 (WRAT4) spelling subtest [37] provided a spelling score to assess general cognitive ability [9], with percentage correct as the dependent variable. In an untimed task, forty two words of increasing difficulty were read out, followed by a sentence for context, with participants writing the word alone on an answer sheet.

#### Mathematical achievement

A timed, paper-based calculation task provided a mathematical achievement index for each participant [38]. The task consisted of addition, subtraction, and multiplication questions, in which participants were asked to answer as many questions as possible by writing their answers after each arithmetical problem. The addition and subtraction sections consisted of a 30 second (s) and 90s subsection, whilst the multiplication section included a 40s and 4 minute subsection, with 25 single and two-digit mathematical problems per subsection. Percentage correct was the dependent variable.

#### Inhibition

The classic colour Stroop [35] and number Stroop tasks [36] measured inhibition. For the colour Stroop, participants read a list of 30 black-ink (neutral) colour-words, followed by naming coloured dots (also neutral) as quickly as possible. Participants then identified the ink colour of incongruent coloured words (e.g. ‘RED’ in blue ink). The Stroop effect was calculated by subtracting mean speed on the neutral tasks from speed on the incongruent task. A number Stroop task was used as a further measure of inhibitory control. The number Stroop (based on [36]) consisted of a numerical and a physical size (henceforth physical) task. In the numerical task, participants chose the side of the screen containing the largest number in numerical magnitude (‘Q’ = left, ‘P’ = right). In the physical task, participants chose the side containing the physically larger number. Arabic digits from 1 to 9 (excluding 5), appeared in the centre of the screen in Courier 27pt (0.6°) and 32pt (0.8°) at a horizontal visual angle of 10°. Digit pairs had a numerical distance of 1, 2, or 5. The practice block contained 36 trials, with the pairs 4–5 and 5–6 in distance bin 1, 3–5 and 5–7 in distance 2, and 4–9 and 1–6 in distance 5. Practice trials included novel pairs, but this was not possible for distance bin 5 (as in [36]). There were 48 trials in each condition: congruent, neutral and incongruent, with 144 trials in total. During a congruent trial, the numerically larger number was also physically the largest (e.g. 4–9). During an incongruent trial, the numerically larger number was physically the smallest (e.g. 4–9). Neutral trials varied by task type: in the physical task, a neutral trial consisted of two identical Arabic digits in different physical sizes (e.g. 3–3). Therefore, all neutral trials in the physical task had a numerical distance of 0. In the numerical task, neutral trials consisted of pairs of different Arabic digits of the same physical size (e.g. 3–7). Although both the physical and numerical tasks consisted of equal numbers of trials in the neutral, congruent, and incongruent conditions, the number of trials in each distance bin per congruency condition varied between the tasks. In the numerical task, there were 16 trials for each distance per congruency condition (16 trials in each of the three distance bins = 48 trials per congruency condition). For the physical task there were also 16 trials for each distance per congruency condition, but due to the distance of 0 for neutral trials, this only applied to congruent and incongruent trials for each distance bin (i.e. 16 trials x 2 congruency conditions = 32 trials per distance bin). It should be noted however that this did not impact upon the interference analyses necessary for the current study. During the task, a fixation (+) first appeared for 300ms, followed by a 500ms delay. Stimuli then appeared for up to 5000ms until response, followed by a 1500ms interstimulus interval preceding the next trial. The dependent variable used for the analyses was the interference effect for the physical and numerical tasks: mean RT on incongruent trials—mean RT on neutral trials.

#### Approximate number system

The Panamath task was used to measure foundational non-symbolic numerical processing, with three dependent variables (*w*, accuracy, and RTs). In a between-subjects design, participants were randomly allocated to either a separated dot display or an intermixed dot display condition. Participants were asked to decide as quickly but as accurately as possible whether they had seen more blue or yellow dots (displayed for 200ms). Each trial was initiated by the participant pressing the space bar. In the intermixed dot display condition, blue and yellow dots (between 5 and 21 per array) appeared on a grey background in intermixed windows (100% overlap on-screen: see Panamath software: http://www.panamath.org/researchers.php, [1], the displays of blue and yellow dots were spatially overlapped on-screen). During the separated dot display condition, blue dots appeared on the right and yellow dots on the left of the screen in windows of 45% (i.e. overlap of blue and yellow dots was not possible due to a gap between arrays of 10% screen size). Participants chose the most numerous dot set using the ‘A’ (yellow) and ‘L’ (blue) keyboard keys covered with correspondingly coloured dots. Trials were either congruent or incongruent. During congruent trials, the more numerous dot set also had the largest cumulative surface area and therefore a larger average dot size. Dot sets in incongruent trials had an equal cumulative surface area resulting in a larger average dot size for the least numerous set. There were eight trial types (2 x congruency, 4 x ratio: 1.1, 1.19, 1.32, 2.28) with 400 trials total.

#### Exclusion measures

The Geriatric Depression Scale (GDS) [39] was used to measure possible depression, with a score >5 leading to exclusion (no participants were excluded from the current study as a result of GDS score). The GDS was used as data were collected concurrently with a group of older participants for further research.

### Procedure

The study used a mixed design, with tasks completed in a partially counterbalanced manner. Firstly, participants were randomly allocated to either a separated or an intermixed dot display condition for the Panamath task. Secondly, participants completed one number Stroop task (either the physical or the numerical task) at the beginning of the session, and the other at the end of the session to avoid carry-over effects, with this order counterbalanced across participants. The order in which participants undertook the remainder of the tasks (GDS, mathematical achievement, spelling, and colour Stroop), including their allocated Panamath condition was counterbalanced. Computerised tasks were viewed from a distance of 57cm, on a 1280x1024 60Hz screen. Panamath ran from Java, and the number Stroop tasks ran from E Prime 2.

## Results

Weber fractions (*w*), accuracy, and reaction times (RTs) as dependent variables of ANS acuity are discussed in terms of congruent and incongruent trials [26,40]. Mixed ANOVAs were conducted for *w*, accuracy, and RTs, with dot display as a between-subjects factor (separate, intermixed), and congruency as a within-subjects factor (congruent, incongruent). Correlations were then investigated between ANS acuity, inhibition, and mathematical achievement, with partial correlations assessing the relationship between ANS acuity and mathematical achievement when controlling inhibition. The raw data were trimmed by applying a 3 SD cut-off for individual RTs (1.91% of data removed). Data for 2 participants were excluded due to *w*_s_ deviating by over 4 SDs from the group mean [24]. One participant was excluded due to a GDS score >5. Finally, 2 participants were excluded for failing to complete the non-symbolic task. A total of 59 participants’ data were analysed, with participant numbers reflecting those of similar studies [10,15,16]. Confidence intervals at 95% are reported.

### Weber Fractions

A 2 (dot display) x 2 (congruency) mixed ANOVA was conducted on *w*_s_. *W*_s_ were significantly higher (poorer ANS acuity) during incongruent (M = .276, CI [.255, .296]) compared to congruent trials (M = .210, CI [.198, .222]; *F*(1,57) = 51.88, *p* < .001, $\eta_{p}^{2}$ = .477). *W*_s_ were also higher for participants in the intermixed (M = .282, CI [.261, .302]) compared to the separate condition (M = .204, CI [.183, .225]; *F*(1,57) = 28.77, *p* < .001, $\eta_{p}^{2}$ = .335). The interaction between congruency and dot display did not reach significance (*p* = .845, $\eta_{p}^{2}$ = .001).

### Accuracy

**Fig 1:**
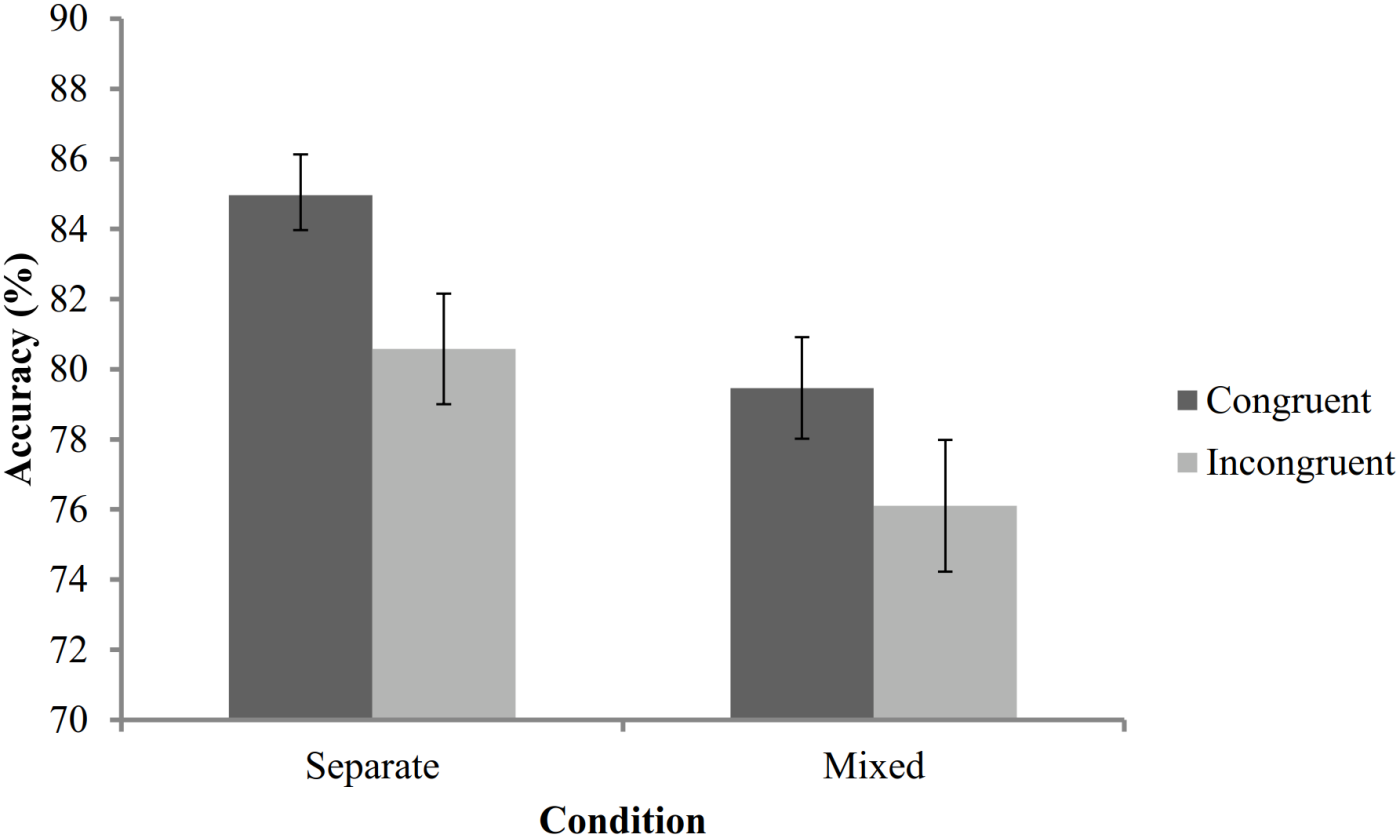
Accuracy (%) during congruent and incongruent trials for intermixed and separate displays (95% confidence intervals).

A 2 (dot display) x 2 (congruency) mixed ANOVA was conducted on accuracy. The results mirror the *w* analyses. Accuracy was lower during incongruent (M = 78.34%, CI [77.14, 79.55]) compared to congruent trials (M = 82.22%, CI [81.31, 83.13]: *F*(1,57) = 45.70, *p* < .001, $\eta_{p}^{2}$ = .445). Participants in the intermixed condition were less accurate (M = 77.79%, CI [76.52, 79.05]) than those in the separate condition (M = 82.77%, CI [81.49, 84.06]; *F*(1,57) = 30.68, *p* < .001, $\eta_{p}^{2}$ = .350). The interaction between congruency and dot display did not reach significance (*p* = .372, $\eta_{p}^{2}$ = .014; see Fig 1).

### RTs

A 2 (dot display) x 2 (congruency) mixed ANOVA was conducted on correct-trial RTs. Participants were significantly slower during incongruent (M = 878.92ms, CI [837.73, 920.10]) compared to congruent trials (M = 850.02ms, CI [812.16, 887.89]: *F*(1,57) = 38.22, *p* < .001, $\eta_{p}^{2}$ = .401). Participants in the intermixed condition were slower (M = 924.93ms, CI [869.85, 980.02]) than those in the separate condition (M = 804.01ms, CI [747.98, 860.03]; *F*(1,57) = 9.50, *p* = .003, $\eta_{p}^{2}$ = .143). The interaction between congruency and dot display did not reach significance (*p* = .519, $\eta_{p}^{2}$ = 007).

### Correlations between ANS acuity, Mathematical Achievement, and Inhibition

An interference effect (RT on incongruent trials—RT on neutral trials) was calculated on correct trials for the numerical and physical number Stroop tasks. Full analysis of the number Stroop can be found in the supporting information (S1 Word). To further investigate the role of inhibitory control during ANS tasks, difference scores between accuracy and RTs for congruent and incongruent trials were calculated for each participant [26]. Holm’s correction was applied to all bivariate correlations to correct for potentially elevated type 1 error (this did not alter the significance status of any correlation).

Firstly, correlations with a two-tailed level of significance were conducted between control measures (years of education, mathematical achievement, spelling, colour Stroop, and number Stroop interference effects) for all participants. Education did not correlate with any measure (*p*_s_ > .1). Superior mathematical achievement was related to a higher spelling score (*r* = .365, *p* = .005), reflecting a relationship between mathematical achievement and verbal abilities [6,23]. Superior mathematical achievement was marginally associated with better inhibitory control in the colour Stroop task (*r* = -.243, *p* = .064). There was also a link between a higher spelling score and lower interference on the numerical number Stroop task (*r* = -.294, *p* = .024). Finally, interference effects from the colour Stroop and physical number Stroop task correlated (*r* = .291, *p* = .027).

**Fig 2:**
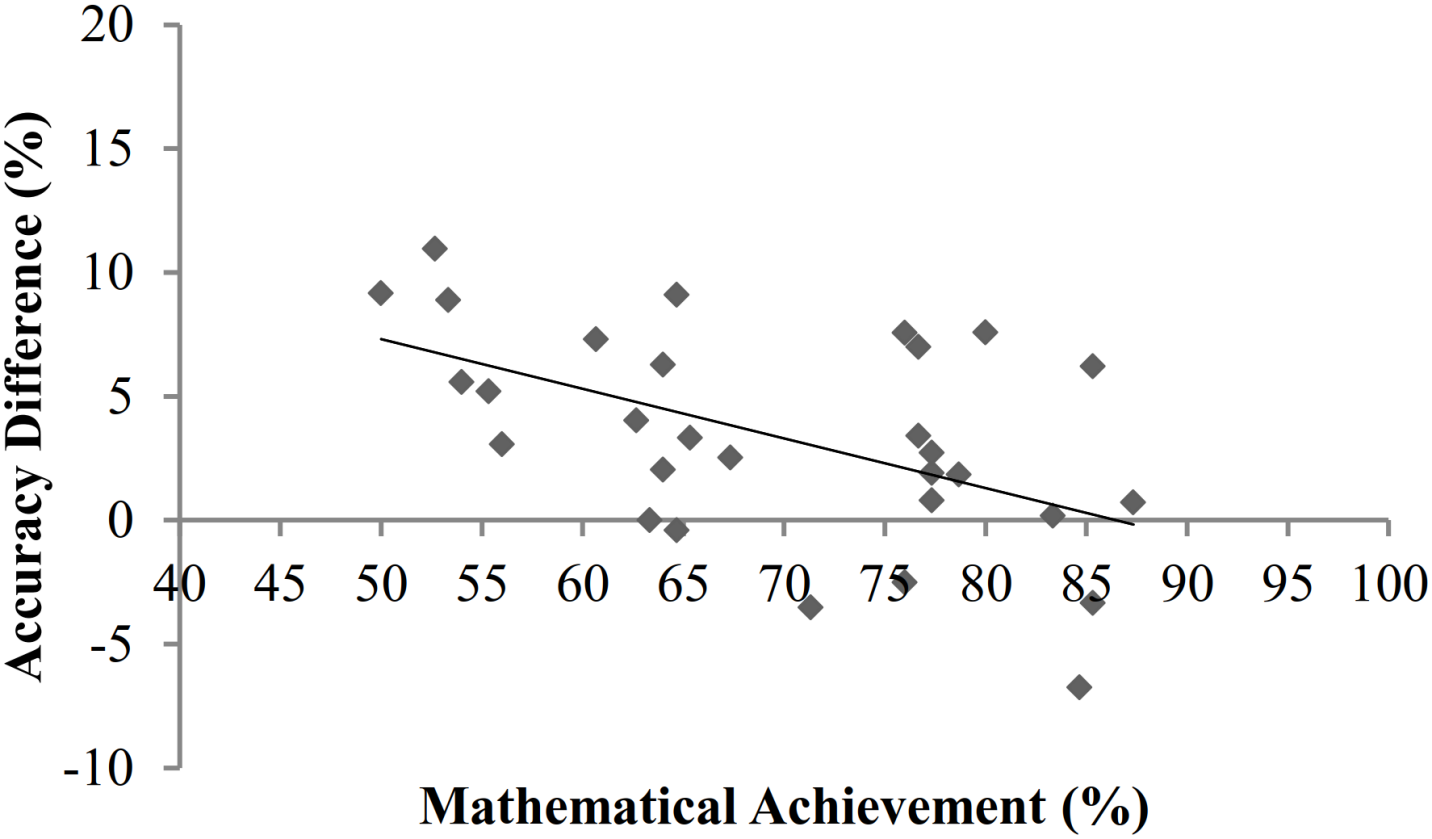
The negative relationship between mathematical achievement and accuracy difference for participants in the intermixed condition (*r* = -.525, *p* = .003).

**Fig 3:**
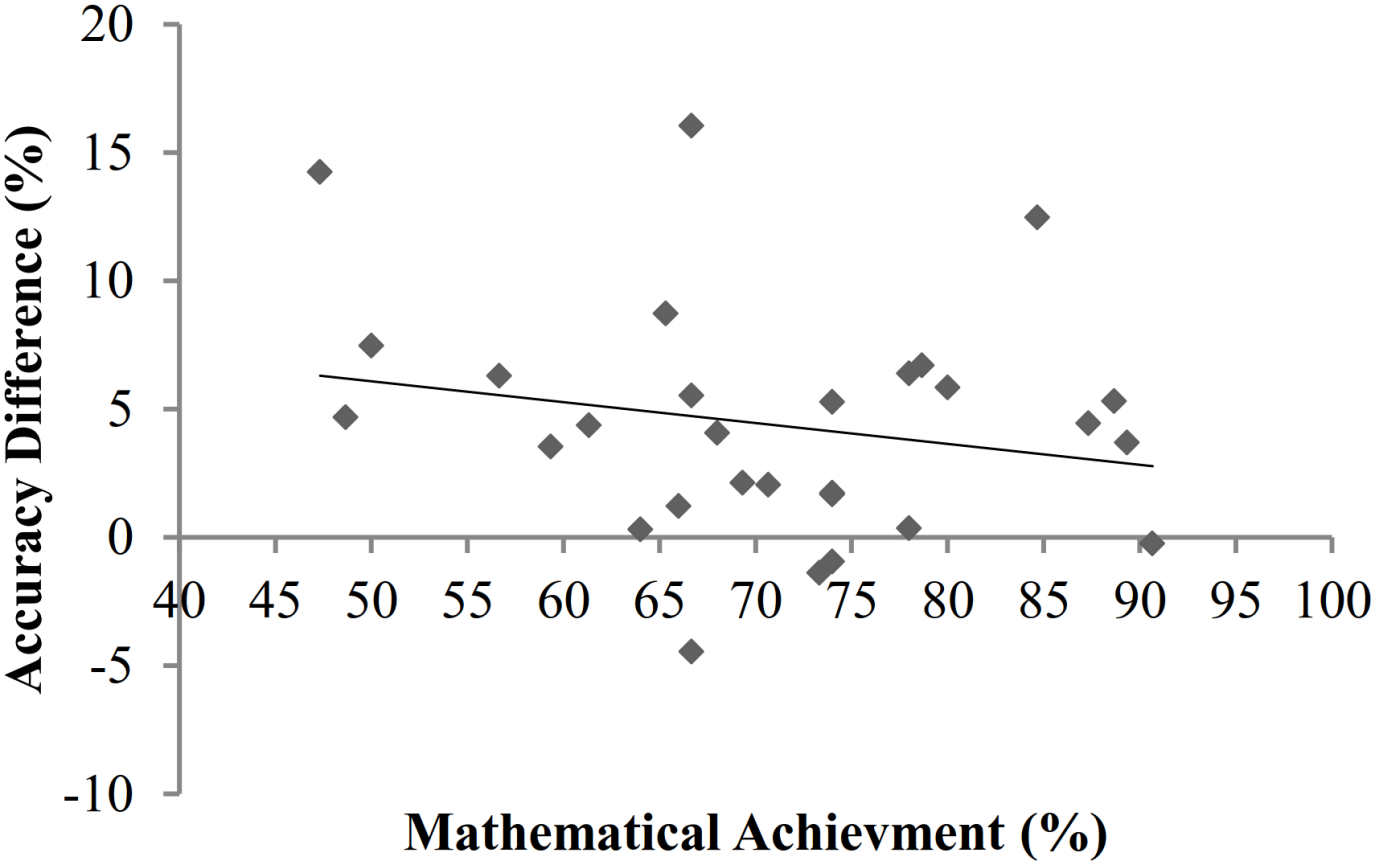
The non-significant relationship between mathematical achievement and accuracy difference for participants in the separate condition (*r* = -.212, *p* = .270).

Secondly, split-half reliabilities were calculated using the Spearman-Brown formula (as in [21]). Data was halved ensuring an equal split of trials per ratio bin and congruency condition in each half. Reliability for all participants’ accuracy scores was relatively low: *r* = .635. The values decreased further when individual analyses were conducted for the separate and intermixed conditions (*r* = .568 and *r* = .444 respectively). Thirdly, correlations between mathematical achievement, inhibition, and ANS acuity were conducted separately for intermixed and separate conditions to investigate the possibility of unique effects of dot display on the ANS—mathematical achievement relationship (see Tables 1 and 2). Studies finding a link between mathematical achievement and ANS acuity have most frequently used intermixed dot displays (e.g. [23,24]). As prior research has implicated inhibitory control in the ANS—mathematical achievement relationship, and stronger inhibition is linked to higher mathematical achievement, correlational analyses will demonstrate whether relationships between mathematical achievement, ANS acuity, and inhibition are mediated by dot display. It should be noted that several significant correlations towards the right tail of each matrix represent relationships between the dependent variables from the ANS task. Table 1 shows that ANS acuity for participants in the separate dots condition did not correlate with mathematical achievement. However, correlations between a larger interference effect on the physical number Stroop task and higher *w*, and between a larger numerical number Stroop interference effect and a larger RT difference (the difference between RTs on congruent and incongruent trials in the ANS task) demonstrate a relationship between interference effects created by congruent vs. incongruent trials in the separate dots ANS condition and inhibitory control. As shown in Table 2, in the intermixed dots condition accuracy difference (the difference between accuracy scores on congruent and incongruent ANS trials) significantly correlated with mathematical achievement. This shows a link between a smaller accuracy difference, reflecting greater inhibitory control (i.e. a smaller ANS interference effect), and higher mathematical achievement scores (see Figs 2 and 3). However, a Fisher’s *r* to *z* transformation revealed that the correlations between accuracy difference and mathematical achievement in the separate and intermixed conditions did not significantly differ (*z* = 1.34, *p* = .18). Further, as in the separate dots condition, there was a relationship between RT difference in the intermixed condition and the numerical number Stroop interference effect (*r* = .542, *p* = .001). These correlations demonstrate a relationship between inhibitory control and performance on non-symbolic comparison tasks, and also indicate that a relationship between the inhibitory component of ANS acuity and mathematical achievement may appear when intermixed dot displays are used. Finally, to further investigate the impact of inhibitory control on the relationship between mathematical achievement and ANS acuity, partial correlations were conducted for the separate and intermixed dot display conditions, controlling for interference effects on the colour and number Stroop tasks. The relationship between accuracy difference and mathematical achievement in the intermixed condition remained significant when controlling inhibition (*r* = -.364, *p* = .006). Moreover, controlling for inhibition did not affect any other correlations.

**Table 1:** Correlations between ANS acuity, mathematical achievement, and inhibitory control for the separated dots condition.

	1	2	3	4	5	6	7	8	9	10	11	12	13	14	15	*M (SD)*
1. Mathematical Achievement	-															*71 (12)*
2. Colour Stroop Interference	-.320	-														*10 (5)*
3. Physical Interference	.102	.364	-													*79 (47)*
4. Numerical Interference	-.168	-.137	-.169	-												*26 (27)*
5. Overall Accuracy	.130	-.178	-.354	-.273	-											*83 (3)*
6. Congruent Accuracy	-.035	-.010	-.290	-.333	.710**	-										*85 (3)*
7. Incongruent Accuracy	.206	-.239	-.273	-.130	.852**	.236	-									*81 (4)*
8. Accuracy Difference	-.212	.212	.053	-.106	-.300	.459*	-.755**	-								*4 (5)*
9. Overall RT	-.200	.109	.012	.111	.094	.223	-.032	.180	-							*804 (134)*
10. Congruent RT	-.219	.135	.031	.067	.089	.232	-.047	.199	.996**	-						*791 (133)*
11. Incongruent RT	-.181	.083	-.007	.154	.096	.213	-.023	.165	.996**	.984**	-					*817 (136)*
12. RT Difference	.188	-.276	-.212	.496**	.051	-.079	.128	-.170	.123	.034	.211	-				*26 (24)*
13. Overall *w*	-.155	.162	.391*	.253	-.985**	-.660**	-.868**	.349	-.081	-.072	-.087	-.095	-			*0*.*20 (0*.*04)*
14. Congruent *w*	.011	-.018	.269	.292	-.673**	-.982**	-.198	-.481**	-.209	-.214	-.205	.022	.639**	-		*0*.*17 (0*.*03)*
15. Incongruent *w*	-.218	.238	.319	.145	-.824**	-.202	-.986**	.765**	.051	.067	.039	-.151	.863**	.166	-	*0*.*24 (0*.*06)*

*. *p* < 0.05 (2-tailed)

**. *p* < 0.01 (2-tailed)

**Table 2:** Correlations between ANS acuity, mathematical achievement, and inhibitory control for the intermixed dots condition.

	1	2	3	4	5	6	7	8	9	10	11	12	13	14	15	*M (SD)*
1. Mathematical Achievement	-															*70 (11)*
2. Colour Stroop Interference	-.158	-														*8 (3)*
3. Physical Interference	.077	.279	-													*87 (58)*
4. Numerical Interference	-.074	-.097	-.249	-												*46 (38)*
5. Overall Accuracy	.157	-.141	-.078	-.207	-											*78 (4)*
6. Congruent Accuracy	-.129	-.074	-.033	-.341	.852**	-										*79 (9)*
7. Incongruent Accuracy	.346	-.169	-.098	-.061	.915**	.567**	-									*76 (5)*
8. Accuracy Difference	-.525**	.132	.086	-.238	-.305	.239	-.664**	-								*3 (4)*
9. Overall RT	-.081	.032	.111	.298	.212	.274	.124	.103	-							*925 (165)*
10. Congruent RT	-.084	.009	.142	.236	.219	.296	.117	.131	.992**	-						*909 (156)*
11. Incongruent RT	-.078	.052	.080	.351	.202	.249	.126	.078	.993**	.972**	-					*941 (177)*
12. RT Difference	-.014	.176	-.177	.565**	.032	-.049	.088	-.148	.463*	.350	.561**	-				*32 (44)*
13. Overall *w*	-.117	.150	.157	.179	-.983**	-.832**	-.904**	.310	-.196	-.201	-.188	-.041	-			*0*.*28 (0*.*07)*
14. Congruent *w*	.098	.039	.091	.345	-.860**	-.982**	-.594**	-.191	-.276	-.293	-.257	.007	.863**	-		*0*.*25 (0*.*06)*
15. Incongruent *w*	-.245	.197	.166	.051	-.908**	-.592**	-.971**	.607**	-.104	-.099	-.104	-.065	.930**	.624**	-	*0*.*32 (0*.*10)*

*. *p* < 0.05 (2-tailed)

**. *p* < 0.01 (2-tailed)

## Discussion

In this study, the effect of dot display on ANS acuity was investigated, with participants completing a numerosity discrimination task with either intermixed or separated dot displays. The focus of the study was on determining how such methodological differences, present but unaccounted for throughout the literature, affect ANS acuity and its link with mathematical achievement. Intermixed dot displays yielded poorer ANS acuity compared to separate displays, contradicting the findings of Price et al. [15] who found stable ANS acuity across intermixed, separate, and sequential displays. Our study differs from that of Price et al. (2012) in crucial ways which may explain the contradictory findings. Firstly, we used a between-subjects design to reduce practice effects, whereas Price et al. used a within-subjects design. Due to the correlational analyses included within the current study, a between-subjects design may be considered a limitation. However, as the research primarily aimed to ascertain without the influence of practice and fatigue effects whether ANS acuity is affected by dot display, a between-subjects design was used. Second, we used a 200ms display [1] to ensure only approximate abilities, and not counting, were measured. Price and colleagues’ 750ms display may have encouraged counting and other strategies, possibly facilitating performance by providing more time for the successful resolution of overlapping dot arrays in the intermixed task. This may have masked ANS acuity differences between dot displays. Finally, we measured three indices of the ANS (*w*, accuracy, and RTs for congruent and incongruent trials), whereas Price et al. (2012) only calculated *w* and NREs, the latter of which has been found to be an unreliable measure of ANS acuity [11]. The current study therefore builds upon research investigating the effect of task design on ANS measurement, using short display times to ensure that approximate abilities were measured.

A significant correlation between mathematical achievement and ANS accuracy difference emerged in the intermixed condition. This suggests that the inhibitory component of the ANS task is more likely to correlate with mathematical achievement when using intermixed displays. These parameters must therefore be considered seriously when assessing the ANS and its relationship with other cognitive abilities, particularly mathematical achievement [11,15,21]. However, as a correlation between mathematical achievement and other measures of ANS acuity (*w*, accuracy, and RTs) was not found, it is difficult to determine from the current findings whether intermixed dot displays do indeed increase the likelihood of finding a relationship between the ANS and mathematical achievement. As the majority of studies finding a link between ANS acuity and mathematical achievement in adults have used intermixed displays [16,20–22], future research is required to further investigate the role of display type in mediating the ANS—mathematical achievement relationship. Moreover, where ANS measures demonstrate low reliabilities, conclusions regarding the relationship between ANS acuity and mathematical achievement must be treated with caution. Indeed, the current findings indicate that this may be particularly pertinent to studies using intermixed dot displays. The findings have significant implications for the debate regarding the foundational role of the ANS in mathematical learning, and for emerging studies investigating the impact of ANS training on mathematical achievement (e.g. [31]). Studies proposing that ANS acuity training may improve mathematical achievement must first investigate other cognitive functions potentially required during an ANS task. Should inhibitory control be required, improved mathematical achievement after ANS training may be due to a relationship between stronger inhibition and better mathematical ability [6,26], rather than a relationship between stronger approximate number skills and mathematical abilities specifically (e.g. in ageing [41]).

Inhibitory control has been cited as a contributory factor in the relationship between mathematical achievement and ANS acuity [19], with some suggesting that the relationship between the ANS and mathematical achievement may be entirely mediated by inhibition [26]. The current results demonstrate a requirement for inhibitory control during both separate and intermixed tasks, although the relationship between mathematical achievement and accuracy difference was exclusively significant for the intermixed task. It appears that intermixed stimuli whereby both arrays overlap, in contrast to clearly distinct arrays in separate displays, may increase the inhibitory load of the ANS task, leading to a stronger ANS—mathematical achievement correlation. Therefore, the relationship between mathematical achievement and ANS acuity may be mediated partially or wholly by inhibitory control [6,19,26]. The current findings also implicate better inhibitory control in higher mathematical achievement, with a marginal correlation between higher mathematical achievement and lower interference on the colour Stroop task. Further, a link between mathematical achievement and spelling ability may be related to stronger inhibitory control [6,26], in that higher achievers have superior inhibition skills. Partial correlations demonstrated that when controlling for inhibition as measured by the colour and number Stroop tasks, the correlation between mathematical achievement and accuracy difference in the intermixed condition remained. This finding may implicate additional types of inhibitory control during an ANS task. Moreover, various measures of inhibitory control demonstrate low correlations (e.g. [42]). It is therefore likely that controlling several different measures of inhibition may differently impact upon the ANS—mathematical achievement relationship. It is possible that inhibitory control may be required to a greater extent during intermixed displays as, due to the overlap between the two numerosities, there may be a requirement to inhibit the processing of one dot array (e.g. yellow) whilst processing the other (e.g. blue). However during separate displays whereby arrays are spatially distinct, the numerosity of each array may be processed simultaneously, without the necessity to inhibit one whilst processing the other. Future research into the relationships between ANS acuity when measured with intermixed displays, inhibitory control, and mathematical achievement would benefit from using a variety of inhibition measures [6] to provide specific inhibitory correlates of mathematical achievement and ANS acuity, further uncovering methodological factors mediating such relationships.

Although the mathematical achievement task used in the current study has been applied in similar research [9,38], our mathematical achievement index and those of other studies may benefit from the inclusion of further mathematical problems (e.g. division). Indeed, variation in the indexes of mathematical achievement used throughout the literature may contribute to contradictory relationships found between mathematical achievement, ANS acuity, and inhibitory control. However, that a direct correlation between mathematical achievement and ANS acuity did not reach significance in the current study reflects a non-significant correlation found in previous research using the same test of mathematical achievement (e.g. [9]). Additionally, investigation of other cognitive factors potentially contributing to the relationship between ANS acuity when measured with intermixed displays and mathematical achievement would be beneficial to future research. For example, speed of processing may provide some explanation for this effect, as participants are required to resolve overlapping dot arrays [10], overcome dot-size conflict (during incongruent trials), and make a numerosity judgement, all within a short timeframe (e.g. 200ms). Participants more resilient to this additional processing step may also have superior cognitive abilities, alongside better inhibitory control, attributes which have been found to correlate with better mathematical achievement (e.g. [6]). However, the precise mechanisms of this effect require further examination.

A final crucial consideration of the current findings is the low split-half reliabilities of both separate and intermixed ANS tasks designed using the Panamath software. Indeed, low test-retest reliabilities have previously been found using Panamath (e.g. [17]). Poor reliability appears to be a common finding for measures of ANS acuity, with low test-retest reliability for *w* in adults [11]. Using measures with low reliability not only undermines confidence in the test’s utility in measuring approximate numerical abilities, but also reduces the likelihood of finding a correlation between ANS acuity and mathematical achievement [12]. The current findings provide further evidence of lower reliability for intermixed compared to separate displays [15,18], calling into question the use of intermixed dot displays to measure ANS acuity. Further, varied reliabilities of ANS tasks across the literature may be caused by the different methods with which data are split before conducting reliability analyses. In order to conduct a split-half reliability test, the dataset must necessarily be split into halves. The manner in which authors decide to split the data may therefore greatly affect coefficients. For example, as non-symbolic numerical comparison paradigms usually consist of at least two within-subjects factors (ratio and perceptual variable control), authors may split the data ensuring an equal split of trials per ratio, trials per perceptual variable control condition, or both (as in the current study). As data-splitting methods are rarely reported, it is unclear to what extent different techniques may lead to varied split-half reliabilities in the literature, as well as low reliabilities found for several studies.

This is to our knowledge the first study to directly compare ANS acuity measured with *w*, accuracy, and RTs derived from non-symbolic comparison tasks using intermixed and separate dot displays to investigate the effect of task design on ANS acuity and its relationship with mathematical achievement. Although the results do not fully support previous findings of a link between ANS acuity and mathematical achievement during intermixed dot displays [24], the findings indicate a significant link between inhibitory load during the intermixed task and mathematical achievement, which in other studies using various measures of ANS acuity and mathematical achievement may drive a correlation between ANS acuity and mathematical achievement. Future research would benefit from extending the current findings by implementing a within-subjects design, using standardised tests of mathematical attainment, and including a broader range of general cognitive ability and inhibitory control measures. Studies employing more stringent perceptual variable control [43] would also facilitate further investigation of the effect of stimulus display on ANS acuity and its link with mathematical achievement. However, the current findings reveal consistently poorer ANS acuity and lower reliability when intermixed dot displays are used, posing important questions for the reliable and valid measurement of the ANS. This study therefore provides the groundwork for future research into the effect of dot display on ANS acuity and its relationship with mathematical achievement and inhibition.

## Supporting Information

S1 Word Number Stroop analyses. (DOCX)
