# Supplementary material for: Dot Display Affects Approximate Number System Acuity and Relationships with Mathematical Achievement and Inhibitory Control
Source: PLoS One. 2016 May 19;11(5):e0155543. doi: 10.1371/journal.pone.0155543 (PMC4873147; doi:10.1371/journal.pone.0155543)
Supplement: S1 Word — (DOCX) [file pone.0155543.s001.docx]

Number Stroop Analyses

Full analyses of the number Stroop data are provided here for further supplementary reading for manuscript ‘Dot Display Affects Approximate Number System Acuity and Relationships with Mathematical Achievement and Inhibitory Control’. For analyses within the manuscript, interference effects were derived from the physical and numerical number Stroop tasks as measures of inhibitory control. This document provides further analysis of the number Stroop data.

Raw data were trimmed by applying a 3 SD cut-off for individual RTs on the physical and numerical tasks (1.74% and 1.64% of the data removed respectively). One participant’s data on the physical task was removed due to low accuracy (~8%). In order to first investigate the impact of task type on the congruency effect, a repeated-measures ANOVA was conducted on RTs from correct trials. There were two within-subjects factors: task type (physical, numerical) and congruency (neutral, congruent, incongruent). Distance was not included in these first analyses as all neutral trials in the physical task were digits of identical numerical magnitude (e.g. 2 and 2) with a distance of 0.

There was a main effect of task type (*F*(1,57) = 64.159, *p* < .001), with overall slower responses in the physical task (M = 710ms, CI [676, 744]) compared to the numerical task (M = 598ms, CI [572, 625]), reflecting stronger automaticity of processing the numerical magnitude than the physical size of Arabic digits [2]. There was a main effect of congruency (*F*(2,114) = 156.728, *p* < .001), with contrasts indicating a significant interference effect (faster responses in the neutral condition: M = 637ms, CI [610, 663], compared to the incongruent condition: M = 696ms, CI [666, 725], *F*(1, 57) = 249.634, *p* < .001). However, a non-significant difference between RTs on neutral and congruent trials (M = 630ms, CI [605, 656]) indicated the absence of a facilitation effect (*p* = .140). Finally, the interaction between task type and congruency was significant (*F*(2,114) = 25.922, *p* < .001). The data in Figure 1 suggest that the interaction may be due to greater interference of numerical magnitude on judgements of physical size compared to the effect of incongruent physical size on numerical magnitude judgements.

Fig 1: The interaction between congruency and task type (95% confidence intervals)

In order to investigate the impact of distance on performance, separate ANOVAs were conducted for the physical and numerical tasks [as in 1]. As all neutral trials in the physical task consisted of Arabic digits of identical numerical magnitude and therefore of a distance of 0, these trials were excluded from the analyses [e.g. 2]. Therefore, two repeated-measures ANOVAs were conducted for the physical and numerical tasks separately. For the numerical task there were two within-subjects factors: congruency (neutral, congruent, incongruent), and distance (1, 2, 5). For the physical task there were also two within-subjects factors, however congruency consisted of two levels (congruent, incongruent), and distance (1, 2, 5).

For the physical task, the main effect of congruency was significant (*F*(1, 57) = 150.694, *p* < .001), with slower responses to incongruent (M = 770ms, CI [732, 808]) compared to congruent trials (M = 673ms, CI [641, 706]). Further, the distance effect was significant (*F*(2, 114) = 4.718, *p* = .011). Contrasts indicated non-significant differences between RTs on distances 1 and 5, and 2 and 5 (*p*_s_ > .1), but slower responses to trials with a distance of 2 (M = 731ms, CI [694, 767]) compared to 1 (M = 712ms, [CI 686, 744]: *F*(1, 57) = 8.327, *p* = .006). Finally, there was an interaction between congruency and distance (*F*(2, 114) = 28.082, *p* < .001). Figure 2 demonstrates that the interaction appears to be driven by slower RTs on incongruent trials as distance increases, whereas RTs on congruent trials remain similar for distances 1 and 2, with faster RTs for distance 5.

Fig 2: The interaction between distance and congruency on the physical task (95% confidence intervals)

For the numerical task, there was a main effect of congruency (*F*(2, 116) = 41.723, *p* < .001), with contrasts indicating a significant interference effect (*F*(1, 58) = 67.647, *p* < .001: neutral M = 591ms, CI [563, 618]; incongruent M = 627ms, CI [598, 657]), but no facilitation (congruent M = 592ms, CI [566, 618]: *p* = .798). Further, there was a distance effect (*F*(1.71, 99.09) = 86.863, *p* < .001), with decreasing RTs with increasing distance (Bin 1 M = 634ms, CI [604, 664]; Bin 2 M = 609ms, CI [581, 637]; Bin 5 M = 567ms, CI [543, 592]: all *p*_s_ < .001) [e.g. 1, 2, 3]. Figure 3 demonstrates that the interaction between congruency and distance (*F*(4, 232) = 17.518, *p* < .001) may be due to similar RTs across distances during neutral trials, but faster RTs with increasing distance for congruent and incongruent trials.

Fig 3: The interaction between distance and congruency on the numerical task (95% confidence intervals)

Some of the current findings, particularly those regarding the congruency effect, somewhat contradict previous results. The congruency effect during the numerical task has been found to consist of both a facilitation and interference effect, whereas congruency effects during the physical task are driven interference only [1, 2, 3]. In the current study using methods similar to those of Cohen Kadosh et al. (2008), we found interference effects (no facilitation) for the effect of physical size on numerical magnitude judgements. In some previous studies, the physical size of the ‘small’ and ‘large’ digits differed by as much as 100%, i.e. the large digit was twice the height of the small digit (e.g. experiment 1: [3]), or by as much as 10mm [2]. In the current study, a visual angle difference of just 0.2⁰ was used between the small and large digits [as in 1]. It is possible therefore that the smaller contrast in physical size between the small and large digits may have affected performance on the physical comparison task, potentially making it more difficult to perceive the physically largest digit, resulting in more effortful and less automatic processing of physical size. Indeed, that a facilitation effect was not found for physical size on numerical magnitude judgments supports this suggestion, i.e. the physically larger size of a congruent digit did not facilitate a faster response. Moreover, in contrast to previous results [2, 3, 4], we found that participants were faster overall to respond during the numerical task than the physical task. Although faster responses to the numerical task have been found in some previous research (e.g. [2]: experiments 1 and 2), in this example participants were comparing a presented digit to a standard of 5, making a comparison with the current findings difficult. However, crucially for the current study, interference effects were found in both tasks, which allowed for the calculation of interference effects to be included within the correlational analyses in the manuscript.

References

1. Cohen Kadosh R, Henik A, Rubinsten O. Are Arabic and verbal numbers processed in different ways? J Exp Psychol Learn Mem Cogn. 2008;34(6):1377–91.

2. Henik A, Tzelgov J. Is three greater than five: The relation between physical and semantic size in comparison tasks. Mem Cognit. 1982 Jul;10(4):389–95.

3. Tzelgov J, Meyer J, Henik A. Automatic and intentional processing of numerical information. J Exp Psychol Learn Mem Cogn. 1992;18(1):166–79.

4. Girelli L, Lucangeli D, Butterworth B. The Development of Automaticity in Accessing Number Magnitude. J Exp Child Psychol. 2000 Jun;76(2):104–22.
